# Socio-demographic, behavioural and cognitive correlates of work-related sitting time in German men and women

**DOI:** 10.1186/1471-2458-14-1259

**Published:** 2014-12-11

**Authors:** Birgit Wallmann-Sperlich, Jens Bucksch, Sven Schneider, Ingo Froboese

**Affiliations:** Institute of Sport Science, Julius-Maximilians-University Würzburg, Judenbühlweg 11, 97082 Würzburg, Germany; Institute of Health Promotion and Clinical Movement Science, German Sport University Cologne, Am Sportpark Müngersdorf 6, 50933 Köln, Germany; Department of Prevention and Health Promotion, School of Public Health, Bielefeld University, 33615 Bielefeld, Germany; WHO Collaborating Centre for Child and Adolescent Health Promotion, School of Public Health, Bielefeld University, Universitätsstraße 25, 33615 Bielefeld, Germany; Medical Faculty Mannheim, Mannheim Institute of Public Health, Social and Preventive Medicine, Heidelberg University, Ludolf-Krehl-Straße 7-11, 68167 Mannheim, Germany

**Keywords:** Physical activity, Sedentary behaviour, Health promotion, Health-determinants, Sitting/standing, Sex, Association, Domain-specific approach, Correlates

## Abstract

**Background:**

Sitting time is ubiquitous for most adults in developed countries and is most prevalent in three domains: in the workplace, during transport and during leisure time. The correlates of prolonged sitting time in workplace settings are not well understood. Therefore, the aim of this study was to examine the gender-specific associations between the socio-demographic, behavioural and cognitive correlates of work-related sitting time.

**Methods:**

A cross-sectional sample of working German adults (n = 1515; 747 men; 43.5 ± 11.0 years) completed questionnaires regarding domain-specific sitting times and physical activity (PA) and answered statements concerning beliefs about sitting. To identify gender-specific correlates of work-related sitting time, we used a series of linear regressions.

**Results:**

The overall median was 2 hours of work-related sitting time/day. Regression analyses showed for men (β = -.43) and for women (β = -.32) that work-related PA was negatively associated with work-related sitting time, but leisure-related PA was not a significant correlate. For women only, transport-related PA (β = -.07) was a negative correlate of work-related sitting time, suggesting increased sitting times during work with decreased PA in transport. Education and income levels were positively associated, and in women only, age (β = -.14) had a negative correlation with work-related sitting time. For both genders, TV-related sitting time was negatively associated with work-related sitting time. The only association with cognitive correlates was found in men for the belief ‘Sitting for long periods does not matter to me’ (β = .10) expressing a more positive attitude towards sitting with increasing sitting durations.

**Conclusions:**

The present findings show that in particular, higher educated men and women as well as young women are high-risk groups to target for reducing prolonged work-related sitting time. In addition, our findings propose considering increasing transport-related PA, especially in women, as well as promoting recreation-related PA in conjunction with efforts to reduce long work-related sitting times.

## Background

There is a growing body of evidence that time spent sitting is an emerging health concern [1]. Current findings have shown that sitting time is consistently associated with an increased risk of all-cause mortality [2, 3] and numerous other negative health conditions such as obesity [4], cardiovascular diseases [5, 6], and type 2 diabetes mellitus [7] as well as various other metabolic risk factors [4, 8]. The common assumption that sufficient moderate-to-vigorous physical activity (MVPA) can compensate for prolonged sitting time must be corrected because sitting time has been found to increase the risk of various negative health outcomes independent of MVPA [2, 9]. However, a recent review showed that the risk of premature all-cause death was attenuated but not diminished by physical activity (PA) levels and was in any case responsible for a substantial population-attributable risk fraction [10]. Nevertheless, recent results from cross-sectional analyses suggest very little association between sitting time and cardio-metabolic biomarkers when total PA is adjusted for [11], indicating that the interplay between sitting time and PA is not completely understood and is thus a sedentary behaviour research priority [12]. The physiology of prolonged sitting and its relationship to health outcomes has not yet been elucidated [13]. One suspected biological mechanism behind the adverse health effects is the following physiological response: through the absence of large muscle group contractions compared e.g., with standing, the lipoprotein lipase is suppressed that is necessary for healthy fat metabolism, and the break-down and use of glucose are reduced, which has been seen in animal models [14]. Both mechanisms could lead to poor metabolic health with a long-term risk of different non-communicable diseases.

Sitting time is ubiquitous for most adults in developed countries and is most prevalent in three domains: in the workplace, during transport and during leisure time. The global tertiarisation of occupations as well as significant alterations in workplace environments and work practices have occurred in recent decades, largely driven by technological innovations such as computers and other labour-saving devices, resulting in sedentary work lives for many [15, 16]. Research from Australia showed that 77% of working hours were spent sitting [17]. Recent literature suggests harmful health consequences for prolonged sitting in workplace environments [16, 18–20], and intervention studies designed to reduce this behaviour achieved the first results that support the assumption that interrupting prolonged sitting time during work is associated with reducing health risks [21–24].

However, the correlates of prolonged sitting time in the workplace setting are not well understood [25]. Overall, studies that examine correlates of sedentary behaviour are in early stages and, in most cases, are limited to overall sitting time [26–28], neglecting specific domains; others often focus on TV viewing [29, 30] or leisure-related sitting time [8, 31, 32]. Hence, the need persists for future research that identifies the individual, social and ecological correlates of sitting time in specific domains [33]. In addition, a gender-specific perspective is warranted based on previous findings that showed distinct gender differences concerning overall sitting time [26] and domain-specific sitting time [32] as well as work-related sitting time [34]. Therefore, the socio-demographic correlates of workplace sitting are of interest for identifying the target groups with the highest need for effective workplace interventions. Separately, individual factors such as habits or attitudes towards prolonged sitting could potentially be associated with individual behavioural choices regarding reducing and reorganising sitting time during work and might be important correlates in future interventions. The first studies that concern leisure behaviour indicate that sedentary behaviours may be intentional and planned from a primary attitude base [35]. Furthermore, it is important to learn more about the behavioural correlates of work-related sitting time. It is, for example, of interest whether MVPA behaviour in the different domains and sitting behaviour in contexts other than work are associated with sitting time during work to support healthy lifestyles, even when compensation for the prolonged sitting time during work cannot be achieved [2].

Therefore, the aims of this study were to examine the gender-specific prevalence of work-related sitting time and to examine gender-specific associations between socio-demographic (i.e., age, education level, income level), behavioural (i.e., work-related PA, travel-related PA, leisure-related PA as well as sitting time during transport, during TV watching, during leisure computer use and during leisure time) as well as cognitive correlates (i.e., health-related beliefs about sitting time) and sitting time in the workplace.

## Methods

### Study design

We conducted a nationwide cross-sectional questionnaire-based study on health behaviours including questions about self-reported sitting time and PA in Germany. Within this scope, the service research centre Growth from Knowledge (GfK) in Nuremberg collected representative data for the distribution of the German population between February and April 2014 as part of a computer-assisted telephone interview (CATI). Pre-tests were conducted in February 2014 during which the selected professional interviewers were trained in administering the computer-assisted standardised questionnaire. All study procedures were approved by the Ethics Committee of the German Sport University in Cologne.

### Study population

In total, 3102 representative residents (1512 men, 1590 women) from the 16 German federal states who were over 18 years of age (mean 50.3 ± 17.7) were interviewed. The sample was taken from the ADM Pool for Telephone Samples (ADM = **A**rbeitskreis der **d**eutschen **M**arkt- und Sozialforschungsinstitute – a study group of German market and social research institutions). The ADM pool is a precisely coordinated national sample based on all possible telephone numbers that forms the basis for selecting population samples in the Federal Republic of Germany. The sample drawing was stratified according to age and gender, and the sample was weighted afterwards to the German population (year 2013) by federal state, residential density and household size according to the data from the National Federal Statistical Office. The overall response rate for the study sample was 13%. Based on the aims of the current study, we only included participants 1) who were 18 to 65 years of age and 2) who were working. Because of these inclusion criteria and our data-cleaning process, we excluded respondents because of age (n = 732), lack of education or employment status (n = 629), missing PA data (n = 127) or missing sitting time data (n = 99). Ultimately, our sample consisted of 1515 participants (747 men; 43.5 ± 11.0 years).

### Measures

#### Sitting time

To assess sitting time, we used the Marshall Sitting Questionnaire [36]. Five items were used to assess time spent in specific sitting pursuits (hours and minutes) each day in the following domains: (a) while travelling to and from places (e.g., work, shops); (b) while at work; (c) while watching television; (d) while using a computer at home; and (e) at leisure not including watching television or using the computer (e.g., visiting friends, movies, eating out) on weekdays and weekend days. The reliability of the instrument has been demonstrated to be moderate across the work, television and computer domains for weekdays (r = 0.78 to 0.84). However, the reliability was weaker for weekend days, except for television (r = 0.57) and computer use (r = 0.74). Validity assessed against log data and sedentary accelerometer data was highest for weekday sitting time at work (r = 0.69) and using a computer at home (r = 0.74) [36]. The Marshall Sitting Questionnaire was translated from English to German. The overall sitting data were considered valid if participants did not report more than 960 minutes of sitting per day (16 hours). As the dependent variable, we focused on sitting time during work on weekdays. All sitting time measures other than work-related on weekdays we considered independent variables.

#### Physical activity

PA was assessed with the Global Physical Activity Questionnaire (GPAQ) [37], which was designed to measure PA in three domains for a typical week: work (paid and unpaid), transport (e.g., walking and cycling to get to and from places), and leisure activities (sports, active living) [37]. Within the work and leisure domains, information on the frequency and duration of both vigorous- and moderate-intensity PA was obtained. For the transport domain, information on all walking and cycling activities was included as moderate-intensity PA. Weekly minutes of moderate- and vigorous-intensity PA were calculated separately by multiplying the number of days per week by the duration of PA on an average day according to [38]. Reported minutes per week in each category were multiplied by the metabolic equivalent (MET; MET-minutes/week^-1^), which is commonly used to express PA intensity independent of body weight. Four METs corresponded to the time spent in moderate-intensity activities and eight METs to the time spent in vigorous-intensity PA. To calculate the average domain-related PA per day, moderately and vigorously intense MET-minutes/week^-1^ were summed (work and leisure) and moderately intense MET-minutes/week^-1^ for transport were computed and divided by seven.

Validity and reliability were assessed previously in nine different countries. Concurrent validity between the International Physical Activity Questionnaire (IPAQ) and the GPAQ showed a moderate to strong positive relationship (range 0.45 to 0.65), and reliability was moderate to good (Kappa 0.67 to 0.73; Spearman’s rho 0.67 to 0.81) [39]. The results for criterion validity using pedometers or accelerometers over the course of seven days were poor to fair (range 0.06 to 0.35). However, the sizes of the correlation coefficients were similar to the ranges reported in other studies [39].

#### Cognitive variables

We asked two statements concerning beliefs about sitting: ‘Sitting for long periods does not matter to me’ and ‘When I sit for hours, I feel uncomfortable’. As response categories, we used a five-point (one to five) rating scale (strongly agree to strongly disagree). To ensure that all items with higher scores would indicate more positive attitudes towards sitting, we recoded the item ‘Sitting for long periods does not matter to me’. The items were developed specifically for this study and were tested for psychometric properties using a standard pilot test.

#### Socio-demographic variables

The demographic variables were self-reported age and gender. Additional socio-demographic variables included education and income levels. Education was categorised into the following levels based on the German school system: ‘no school graduation’, ‘10 years of education’, ‘12 years of education’, ‘13 years of education’ and ‘first university degree or higher’. Household net income per month was assessed in nine categories and summarised in three groups based on tertiles: ‘low income’ (< 1500€), ‘middle income’ (1500€–2499€), and ‘high income’ (€ > 2500€).

### Statistical analysis

Gender-specific medians as well as quartiles were calculated for work-related sitting time during weekdays regarding socio-demographic and cognitive correlates. Differences between the genders in work-related sitting time during weekdays were calculated with non-parametric tests. The sample distribution of the dependent variable ‘work-related sitting time’ was positively skewed (skewness = 0.61). Different transformations [40] did not improve the normality of the distribution, and therefore, we did not transform this variable. Before conducting multiple linear regressions, we explored multicollinearity by using a bivariate correlation matrix for all included variables and by computing the variance inflation factors between all pairs of the independent variables to preclude multicollinearity. We did not observe any problems with multicollinearity (variance inflation factor < 2; bivariate correlation not higher than 0.45) [40]. Referring to an ecological approach to sedentary behaviour [25, 33], we executed gender-specific multiple linear regression analyses by the forced entry method to explore the associations between the dependent variable ‘work-related sitting time’ and the behavioural (sitting and PA), socio-demographic, and cognitive variables (Models 1–4) to avoid commingling between the different variables. The socio-demographic variables were age (continuous variable), education level (four categories) and income level (three categories), and the behaviour variables were PA MET-minutes/day^-1^ in the work, transport and leisure (continuous variable) domains and sitting time (minutes/day^-1^) in the transport, TV, computer and leisure (continuous variable) domains. The fourth model included the two cognitive variables (five categories). Statistical significance was set at a level of 0.05. All analyses were conducted using IBM SPSS Statistics 22 for Windows.

## Results

Participants reported a median sitting time during work of 2 hours (120 minutes), which accounted for 31.0 ± 24.9% of overall sitting time with no difference in genders. The age group 30–45 years had the longest sitting times at work among both men and women. Overall, the median work-related sitting time increased from 1 hour for 10 years of education (22.5 ± 23.5%) to 5 hours (45.2 ± 22.4%) for participants with university degrees. Concerning the cognitive variables, we found one gender-specific difference: women who agreed with the belief ‘When I sit for hours, I feel uncomfortable’ (see Table 1) had longer work-related sitting times.

**Table 1:** Work-related sitting time during weekdays, for all and stratified by gender (* = p < .05)

	All (n = 1515)	Men (n = 747)	Women (n = 768)	
	Work-related sitting time (min/day) median (25th percentile; 75th percentile)	Work-related sitting time (min/day) median (25th percentile; 75th percentile)	Work-related sitting time (min/day) median (25th percentile; 75th percentile)	p-value between gender
**All**	120 (28; 300) (n = 1515)	120 (30; 300) (n = 747)	150 (15; 300) (n = 768)	.66
**Age**				
18–29 years	120 (30; 360) (n = 225)	120 (30; 270) (n = 142)	240 (0; 372) (n = 83)	.10
30–45 years	180 (30; 360) (n = 573)	179 (30; 390) (n = 249)	180 (25; 300) (n = 324)	.44
46–65 years	120 (7; 270) (n = 717)	120 (3; 300) (n = 356)	120 (10; 240) (n = 361)	.85
**Education level**				
No graduation	0 (0; 19) (n = 6)	150 (n = 1)	0 (0; 0) (n = 5)	.03*
10 years	60 (0; 240) (n = 416)	30 (0; 120) (n = 209)	60 (0; 240) (n = 207)	.06
12 years	120 (30; 270) (n = 584)	120 (30; 240) (n = 260)	120 (12; 300) (n = 323)	.73
13 years	180 (30; 360) (n = 218)	180 (20; 360) (n = 115)	180 (59; 360) (n = 104)	.18
University degree	300 (120; 420) (n = 279)	300 (123; 420) (n = 150)	240 (113; 420) (n = 129)	.49
**Income groups** Household net income/month				
<1500€	60 (0; 198) (n = 317)	60 (0; 180) (n = 130)	59 (0; 240) (n = 188)	.60
1500–2499€	120 (16; 300) (n = 518)	120 (15; 300) (n = 260)	179 (22; 300) (n = 258)	.20
>2.500€	180 (60; 360) (n = 531)	180 (60; 360) (n = 292)	180 (60; 360) (n = 239)	.86
**Beliefs:** ‘Sitting for long periods does not matter to me’				
Strongly disagree	60 (0; 240) (n = 348)	60 (11; 180) (n = 131)	90 (0; 300) (n = 217)	.20
Disagree	180 (30; 360) (n = 314)	179 (24; 300) (n = 162)	180 (60; 360) (n = 151)	.16
Undecided	180 (15; 300) (n = 352)	180 (30; 331) (n = 172)	180 (5; 300) (n = 181)	.63
Agree	180 (30; 339) (n = 247)	120 (30; 300) (n = 155)	225 (30; 360) (n = 92)	.30
Strongly agree	120 (5; 300) (n = 255)	120 (29; 365) (n = 126)	120 (5; 240) (n = 129)	.21
**Beliefs:** ‘When I sit for hours, I feel uncomfortable’				
Strongly agree	120 (5; 240) (n = 641)	120 (30; 240) (n = 256)	94 (0; 240) (n = 385)	.34
Agree	180 (30; 360) (n = 343)	180 (15; 360) (n = 187)	240 (60; 383) (n = 157)	.01*
Undecided	180 (51; 300) (n = 234)	180 (30; 300) (n = 129)	148 (60; 300) (n = 105)	.78
Disagree	60 (0; 300) (n = 158)	60 (0; 360) (n = 93)	98 (15; 240) (n = 64)	.52
Strongly disagree	120 (5; 299) (n = 133)	76 (5; 384) (n = 81)	180 (5; 240) (n = 53)	.81

Bivariate correlations of the sitting- and PA-related behavioural variables were calculated (data not shown), and most correlations were small. The highest correlations were observed between work-related sitting time and work-related PA MET-minutes/day^-1^ with r = -.422 (p < .001) in men and r = -.321 (p < .001) in women.

Multiple linear regression analyses in men showed that model 1 with the PA-related behavioural correlates (18%) and model 3 with the socio-demographic correlates (13%) explained most of the variance (R^2^) in work-related sitting times (see Table 2). Concerning PA behaviour, we found that sitting time during work was negatively correlated with work-related PA (β = -.43), suggesting increased sitting durations with less PA during work. Leisure-time physical activity was not a significant correlate. ‘Education’ (β = .29) and ‘income’ (β = .17) were both positively associated with ‘sitting time during work’, that is, increased education and income levels were positively associated with longer sitting times. Model 2 showed that TV-related sitting time (β = -.16) was negatively associated with work-related sitting time. In model 4, the belief ‘Sitting for long periods does not matter to me’ (recoded) (β = .10) was positively correlated with work-related sitting time, reflecting more positive attitudes towards sitting with increasing sitting durations.

**Table 2:** **Results from the multiple linear regressions on the contributions of the socio-demographic, behavioural and cognitive correlates to the dependant variable “sitting time during work” for men**

	Model 1 (n = 747)				Model 2 (n = 722)				Model 3 (n = 682)				Model 4 (n = 746)			
	Behavioural correlates (PA)				Behavioural correlates (sitting)				Socio-demographic correlates				Cognitive correlates			
	B	SE B	β	P	B	SE B	β	p	B	SE B	β	p	B	SE B	β	p
Work-related PA MET minutes/day^-1^	-.306	.024	-.425	≤.001***												
Transport-related PA^-1^	.069	.094	.025	.461												
MET-minutes/day																
Leisure-related PA	-.120	.127	-.032	.346												
MET-minutes/day^-1^																
Sitting time transport					.035	.106	.028	.768								
Sitting time TV					-.382	.090	-.156	<.001***								
Sitting time computer					.166	.112	.058	.137								
Sitting time leisure					-.113	.095	-.046	.234								
Age									.582	.516	-.041	.260				
Education level									35.348	4.430	.294	≤.001***				
Income level									37.777	8.106	.170	≤.001***				
‘Sitting for long periods does not matter to me’ ^b^													12.801	4.726	.104	.007**
‘When I sit for hours, I feel uncomfortable’ ^a^													-2.527	4.710	-.021	.592
*R* ^*2*^	0.179				0.009				.130				.010			

^a^Response options: strongly agree (1), somewhat agree (2), in between (3), somewhat disagree (4), strongly disagree (5).

^b^Response options were recorded as: strongly disagree (1), somewhat disagree (2), in between (3), somewhat agree (4), strongly agree (5).

(B = unstandardised beta; SE B = standard error of beta; β = standardised beta; ** = p < .01; *** = p < .001).

Among women, multiple linear regression also showed the highest explained variance in models 1 (11%) and 3 (10%) (see Table 3). The PA-behavioural correlates showed that work-related (β = -.32) and transport-related PA (β = -.07) were both negatively associated with work-related sitting time; sitting time during work increased with decreased PA during transport and work. Leisure-related PA was also not associated with work-related sitting time. Concerning socio-demographics, age (β = -.14) was negatively correlated with work-related sitting time, showing longer work-related sitting times in younger women. ‘Education’ (β = .21) and ‘income’ (β = .13) were both positively associated with ‘sitting time during work’, demonstrating that education and income levels increased with increased sitting time during work. Model 2, with sitting-related behaviour correlates, found negative associations with TV-related sitting time (β = -.16), that is, decreased TV sitting time with increased sitting time during work. For the cognitive variables, we found no associations.

**Table 3:** **Results from the multiple linear regressions on the contributions of the socio-demographic, behavioural and cognitive correlates to the dependant variable “sitting time during work” for women**

	Model 1 (n = 769)				Model 2 (n = 746)				Model 3 (n = 684)				Model 4 (n = 764)			
	Behavioural correlates (PA)				Behavioural correlates (sitting)				Socio-demographic correlates				Cognitive correlates			
	B	SE B	β	p	B	SE B	β	p	B	SE B	β	p	B	SE B	β	p
Work-related PA MET-minutes/day^-1^	-.330	.035	-.324	≤.001***												
Transport-related PA	-.163	.077	-.073	.034*												
MET-minutes/day^-1^																
Leisure-related PA	.112	.135	.028	.410												
MET-minutes/day^-1^																
Sitting time transport					.103	.135	.028	.443								
Sitting time TV					-.382	.090	-.156	≤.001***								
Sitting time computer					-.052	.093	-.020	.579								
Sitting time leisure					-.161	.093	-.063	.083								
Age									-2.254	.610	-.138	≤.001***				
Education level									33.019	6.279	.205	≤.001***				
Income level									27.690	8.297	.128	.001**				
‘Sitting for long periods does not matter to me’ ^b^													-0.216	4.368	-.002	.961
‘When I sit for hours, I feel uncomfortable’ ^a^													3.201	4.900	.024	.514
*R* ^*2*^	.109				.032				.100				.001			

^a^Response options: strongly agree (1), somewhat agree (2), in between (3), somewhat disagree (4), strongly disagree (5).

^b^Response options were recorded as: strongly disagree (1), somewhat disagree (2), in between (3), somewhat agree (4), strongly agree.

(B = unstandardised beta; SE B = standard error of beta; β = standardised beta; * = p < .05; ** = p < .01; *** = p < .001).

## Discussion

The results showed socio-demographic differences in work-related sitting times, finding a high prevalence of work-related sitting time, with a mean of 5 hours per day, for participants with university degrees. From a behavioural perspective, the most important finding is that neither men nor women showed any association with leisure-related PA; thus, we cannot assume any attempts to compensate for long work-related sitting hours during leisure time. Because both behaviours showed independent relationships with health outcomes, participants tended to show two health-related risk factors [31, 41]. Concerning the additional correlates, we detected gender-specific differences in the behavioural, socio-demographic and cognitive correlates that can inform future interventions to reduce sitting time in workplace settings.

### Prevalence

The median work-related sitting time in the German working population of 2 hours per day, and a mean proportion of roughly 31% of overall sitting time are lower than the findings concerning work-related sitting time from recent studies [42–44]. This may be because the present study does not distinguish between full- and part-time workers and calculates the mean work-related sitting time, which could lead to a bias. In Germany, 27% of the working population works part-time [45]. Data from Australia derived by accelerometers, reflecting approximately 6 hours per day of work-related sitting time, are considerably higher than in our sample [17], which, however, can be explained by the convenience sample of employees in offices, call centres and customer service organisations, which neglected other working groups such as (skilled) workers. Furthermore, the use of objective measurements has been associated with more sedentary behaviour than is self-reported, which mainly reflects the higher sensitivity of objective measures of sitting time and which overcomes the issues of recall bias in self-reported measures [46]. Our findings also showed that a large portion of sitting (one-third) took place at work, but, in contrast, most sitting time was outside the workplace, which was similar to the findings from the Netherlands [43]. This suggests that work-related sitting is only one domain in which to intervene and that the other domains such as leisure, transport etc. need to be considered separately to cover all relevant sedentary behaviours.

We did not observe gender-dependent differences in the work-related sitting times among the working population, which is in contrast with results from Australia that showed significantly more sitting for men [47] but is in accordance with the findings from Mielke and colleagues [42]. Our results suggest a minor role of gender in work-related sitting time in Germany and recommend workplace interventions to reduce sitting times in both genders.

### Correlates of work-related sitting time

Within the aim to investigate separate, gender-specific associations between socio-demographic, behavioural and cognitive correlates and work-related sitting time, we identified the highest explained variances for men and women in the model, including PA-specific behavioural correlates. As was expected, work-related PA was negatively associated with work-related sitting time, which can be easily explained through the “either … or” principle concerning the demands of working posture. Transport-related PA was negatively associated with work-related sitting time only for women, showing that women use active transportation less often when they have long work-related sitting times. This resembles the findings of Chau and colleagues [41] as well as those of Duncan and colleagues [48], who demonstrated that white-collar employees who engage in longer work-related sitting times were less likely to engage in active transportation. However, these findings did not differentiate between men and women. A gender-specific explanation could be that women with children who are enrolled in facilities outside of the home and who work outside of the home are often also responsible for picking up their children from nursery school, school, etc., which can require a vehicle to save time. From a public health perspective, our results advise developing interventions that enable active transportation for the part of the working population that is involved in organising family matters around travel to work.

The present results revealed no association between leisure-related PA and work-related sitting time in either gender, which is in accordance with the findings of [47, 49, 50]. The present results suggest that participants did not attempt to compensate for long sitting durations during work with more PA in their leisure time, which could result in a double health risk [2, 9]. Accordingly, our findings suggest that it might be important to consider both increasing leisure-related PA and reducing work-related sitting time when considering strategies to promote healthy lifestyles in the working population.

Additionally, our results displayed a negative association for men as well as for women between work-related and TV-related sitting time. These findings suggest that people with long sitting durations during work attempt to compensate for their occupational sitting, which is in contrast with [41, 43, 51] but is underlined by national findings that show that higher educated populations also have the most leisure-related PA compared with the lower educated populations [52]. However, this finding could also be attributable to measurement issues [46, 53], i.e., because participants may have compensated with other leisure-related sitting activities that they were not specifically asked about and consequently did not remember, they may have neglected to report these other leisure-related sitting activities. Thus, an additional objective measurement of sitting time would overcome this issue.

The analyses of socio-demographic correlates are of special importance in terms of identifying the target groups that most need interventions. Our study revealed that only in women did age correlate inversely with work-related sitting-time. Evidence from other studies concerning work-related sitting time is rare. However, one explanation for this finding could be in women’s emancipation in Germany, which led to a higher proportion of women with higher education and women working in the third sector, which is especially notable in the younger age groups [54] and would lead to longer work-related sitting times in younger compared with older women. Based on this preliminary evidence, younger age groups should be targeted in interventions to reduce work-related sitting times.

Other important potential socio-demographic correlates of work-related sitting time are education level and income level. A number of studies underlined that education level was positively associated with sitting time at work [42, 43, 55], which was confirmed by our own results for men as well as for women and which indicates that reasonable interventions to reduce work-related sitting time must be developed, especially in tertiary job settings, in which the higher educated population in particular tends to work. This is especially against the background that in these populations, the present study found nearly 50% of the overall sitting time in the working domain. The same findings apply to the results for the positive correlation between income level and work-related sitting time, which is most likely associated with education level and working position. For example, findings from the Netherlands showed distinct differences between occupation sectors, such that people working in computers, commercial services, transportation, banking and insurance, and government and judicial organisations sat for significantly longer than did the average worker [43]. Nevertheless, we did not assess the type of occupation in the present study, which could potentially have confounded the present results for education and income level considering that not all participants with lower education or income levels necessarily work in blue-collar occupations and vice versa. Consequently, the type of occupation should be addressed in future studies because we can assume that sitting time varies as a function of the kind of work people do (e.g., manual vs. non-manual), and workplace interventions to reduce sitting times should focus on jobs for which sitting is the predominate posture. For other working fields such as manual labour, interventions to reduce sitting time should focus on the domains other than work.

The potential cognitive correlates of work-related sitting time can be classified as modifiable factors that could be used as change objectives in interventions. Regarding the cognitive correlates, we only found a positive significant correlation in men with the belief ‘Sitting for long periods does not matter to me’, which shows that behaviour, in this case work-related sitting behaviour and cognition, trend in similar directions and suggests that it would be advisable to work with men on changing their positive beliefs about the benefits of sitting in the work context. In women, we found no associations, which could have been attributable to methodological issues, in particular to the item choices. Research on validated and specific cognitive correlates and sitting time, especially work-related sitting time, such as self-efficacy, perceived behavioural control, and social norms - as was planned in a study in Australia [56] - is lacking to date [57]. This suggests the immediate need for studies that widen their focus beyond socio-demographic and behavioural correlates so that interventions can be developed that consider specific psycho-cognitive determinants. However, it should be kept in mind that sitting behaviour might be often free of conscious processing and have a strong habitual component. Future studies should examine habit strength as a potential correlate along with other variables that explain the intention to sit [58]. Because habits are cued relatively directly by the environment, additional studies also should take an environmental approach to explaining sitting time [25].

### Limitations and strengths

Strengths of this study include the reasonably large sample size and the domain-specific approach concerning sitting and PA behaviour as well as the inclusion of correlates of multiple domains in terms of understanding work-related sitting time, which is unique. Nevertheless, the low response rate of 13% is a limitation. This could have potentially been caused by the overall mean length of (more than 23 minutes of the survey), which covers a broad range of health topics, which could have resulted in a high drop-out rate. Considering the methodology-related literature on surveys [59], the present response rate still seems acceptable for investigating the stated research question. Furthermore, slight deviations from the nationwide population structure caused by the non-responders were balanced out by a weighting procedure. An additional limitation of the study is the missing distinction between part-time and full-time working positions, which would have brought additional useful insights into the specific correlates of working individuals, as well as time spent in the workplace. Moreover, information on the type of occupation is lacking that would have recognised the character of the work being performed. Both should be considered in future research. Furthermore, our information on sitting time was assessed by self-report. Consequently, our results might have been biased owing to misclassifications or social desirability bias. Future research should use both objective and subjective assessments of sitting time to capture important domain- and behaviour-specific sitting time information and to objectively measure total sitting times as well as patterns of sitting [46]. As stated before, the use of psychological scales with reasonable psychometric properties for investigating cognitive correlates should be considered in future research. Furthermore, it should be kept in mind that this study follows a cross-sectional design, which prevents inferences about causality. For example, we cannot determine whether being sedentary at work leads to being more sedentary outside of working hours. Longitudinal research that also acknowledges life-stage issues [33], i.e., the transition between education and working life, to explore the long-term relationships between sedentary behaviour during and outside of working hours and their cognitive and/or environmental correlates could give meaningful insights.

## Conclusion

The present study gives initial insights into the gender-specific socio-demographic, behavioural and cognitive correlates of work-related sitting in the working German population. The present findings showed that in particular, higher educated men and women as well as young women need special attention when developing interventions to reduce prolonged work-related sitting times. Furthermore, our findings suggest considering increases in transport-related PA, especially in women, as well as promoting leisure-related PA for populations with long work-related sitting durations. Only weak associations with the cognitive correlates were found. Here, future research needs to address the specificity of psychosocial as well as environmental correlates and possible associations with specific domains of sitting to obtain more fundamental insights into these associations.

## Abbreviations

PA: Physical activity
MVPA: Moderate-to-vigorous physical activity
ADM: Arbeitskreis der deutschen Markt- und Sozialforschungsinstitute
GPAQ: Global physical activity questionnaire
IPAQ: International physical activity questionnaire.
